# Advances in genomics for diversity studies and trait improvement in temperate fruit and nut crops under changing climatic scenarios

**DOI:** 10.3389/fpls.2022.1048217

**Published:** 2023-01-19

**Authors:** Ikra Manzoor, Kajal Samantara, Momin Showkat Bhat, Iqra Farooq, Khalid Mushtaq Bhat, Mohammad Amin Mir, Shabir Hussain Wani

**Affiliations:** ^1^ Division of Fruit Science, Faculty of Horticulture, Sher-e-Kashmir University of Agricultural Sciences and Technology of Kashmir, Srinagar, India; ^2^ Department of Genetics and Plant Breeding, Institute of Agricultural Sciences, Siksha ‘O’ Anusandhan (Deemed to be University), Bhubaneswar, Odisha, India; ^3^ Division of Floriculture and Landscape Architecture, Faculty of Horticulture, Sher-e-Kashmir University of Agricultural Sciences and Technology of Kashmir, Srinagar, India; ^4^ Field Station Bonera, Pulwama, Council of Industrial and Scientific Research (CSIR) Indian Institute of Integrative Medicine, J&K, Jammu, India; ^5^ Ambri Apple Research Centre, Sher-e-Kashmir University of Agricultural Sciences and Technology of Kashmir, Shopian, India; ^6^ Mountain Research Centre for Field Crops, Sher-e-Kashmir University of Agricultural Sciences and Technology of Kashmir, Jammu and Kashmir, Anantnag, India

**Keywords:** gene mapping, genome sequencing, gene tagging, metabolomics, micro-satellites, molecular markers, QTL, transcriptomics

## Abstract

Genetic improvement of temperate fruit and nut crops through conventional breeding methods is not sufficient alone due to its extreme time-consuming, cost-intensive, and hard-to-handle approach. Again, few other constraints that are associated with these species, *viz*., their long juvenile period, high heterozygosity, sterility, presence of sexual incompatibility, polyploidy, etc., make their selection and improvement process more complicated. Therefore, to promote precise and accurate selection of plants based on their genotypes, supplement of advanced biotechnological tools, *viz*., molecular marker approaches along with traditional breeding methods, is highly required in these species. Different markers, especially the molecular ones, enable direct selection of genomic regions governing the trait of interest such as high quality, yield, and resistance to abiotic and biotic stresses instead of the trait itself, thus saving the overall time and space and helping screen fruit quality and other related desired traits at early stages. The availability of molecular markers like SNP (single-nucleotide polymorphism), DArT (Diversity Arrays Technology) markers, and dense molecular genetic maps in crop plants, including fruit and nut crops, led to a revelation of facts from genetic markers, thus assisting in precise line selection. This review highlighted several aspects of the molecular marker approach that opens up tremendous possibilities to reveal valuable information about genetic diversity and phylogeny to boost the efficacy of selection in temperate fruit crops through genome sequencing and thus cultivar improvement with respect to adaptability and biotic and abiotic stress resistance in temperate fruit and nut species.

## Introduction

Traditional methods of genetic improvement of temperate fruit and nut crops that are generally propagated asexually face a dozen of problems which include a long juvenile phase, absence of seed, higher degree of intra- and interspecific incompatibility, frequent heterozygosity, sterile seeds, and particularly presence of certain specific traits in wild species only (Mehlenbacher, 1995). Natural processes and older tools for generating novel varieties or cultivars of plant species are being used in conventional/classical breeding techniques which are time-consuming, are labor-intensive, and often have less efficiency (Jain and Kharkwal, 2004). The term “molecular markers” refers to naturally occurring polymorphisms in nucleic acids (Thottappilly et al., 2000). A particular segment of the DNA (deoxyribonucleic acid) which represents variations at the genome levels comprises a molecular marker (Dhutmal et al., 2018). At the DNA level, polymorphisms are revealed by molecular markers and might be applied in many genetic studies. A gene or DNA sequence that has a well-known location on a chromosome and is linked to a specific gene or trait is called a genetic marker. It can be characterized as a variance that may result from a mutation or other noticeable modification in the genetic locus (Al-Samarai and Al-Kazaz, 2015). The development of high-throughput molecular markers and genetic maps facilitated the location of genes governing the agronomically important traits and may possibly help in boosting the breeding through MAS (marker-assisted selection). As compared with traditional breeding approaches, MAS is capable of surpassing the obscurity of phenotypic selection including significant efficiency of selection (Wani et al., 2022). A primary gene or several other genes governing an individual trait that is distributed in multiple chromosomal regions are known as quantitative trait loci (QTLs) (Igarashi et al., 2015). Molecular markers can be found at the position of a particular gene in prominent locations of the genome mapping (Raza et al., 2015). Molecular markers which are actually the nucleotide sequences are generally differentiated on the basis of polymorphism or variability present between these nucleotide sequences of different living beings (Nadeem et al., 2018). With the help of genetic marker patterns of heredity, variations in the genome, evolutionary analysis, and marker-assisted breeding can be done for the improvement of different crop species (Hayward et al., 2015).

The role of molecular markers for the characterization and improvement of various genotypic and phenotypic traits in temperate fruit and nut crops such as assessment of genetic diversity in almond, red skin color in apple, sharka disease resistance in apricot, and genome sequencing in almond has been extensively studied in the last few years (Lozano et al., 2014; Halasz et al., 2019; Mori et al., 2019; Alioto et al., 2020). Therefore, to overcome such difficulties, the implementation of radical and sophisticated tools of molecular breeding along with classical breeding would be an efficient and reliable alternative. The molecular marker approach is a viable option which selects desirable individuals in a breeding program based on the use of DNA markers rather than, or in addition, to their trait values. This review provides facts regarding several applications of molecular marker and omics approaches in temperate fruit and nut crop breeding in terms of their genetic improvement and cultivar development.

## Genetic diversity assessment

Recent advances in molecular markers propose great possibilities to assess the genetic diversity in a vast range of germplasms (Nawaz et al., 2013; Nawaz et al., 2016; Wang et al., 2017). Genetic diversity estimation is very useful to study the plant evolution and in turn the comparative genomics of plants, for understanding the composition and construction of various populations (Liu et al., 2015; Nawaz et al., 2017). In recent years, a lot of research has been done for the assessment of genetic diversity in temperate fruit and nut crops *via* the molecular marker approaches (**Table 1**). Thurow et al. (2016) reported that a population genetic assessment of Brazilian peach breeding germplasm was determined using SSR (simple sequence repeat) markers, selected by their high polymorphism levels. This study was conducted in 204 desirable peach genotypes and is the first acumen of available peach genetic variability. Later studies laid the evidence of the population structure of peach to be exploited facilitating the genome-wide similarity and relationship studies. Lim et al. (2016) conducted a study using 5SSR markers that are highly polymorphic in nature, to report the genetic diversity among 160 strawberry accessions. They discovered 60 distinct alleles, with allele frequencies ranging from 0.006 to 1 and similarity ratings ranging from 0.034 to 0.963 (average: 0.507). There was frequent clubbing of accessions within a pedigree, and further study revealed a total of 30 unique accessions classified along with the existing accessions, allowing fruit breeders to develop strategies in order to assess genetic diversity present in the new cultivars.

**Table 1:** DNA markers for genetic diversity assessment in temperate fruit and nut crops.

Fruit/nut crop	Molecular marker types	References
Peach (*Prunus persica* L. Batsch.)	SSR	Thurow et al. (2016)
Strawberry (*Fragaria × ananassa* Duch.)	SSR	Lim et al. (2016)
Sandy pear (*Pyrus pyrifolia* Nakai.)	SSR	Jan (2016)
Hazelnut (*Corylus avellana*)	AFLP (amplified fragment length polymorphism) and SSR	Öztürk et al. (2017)
Kiwifruit (*Actinidia* spp.)	SNP (single-nucleotide polymorphism)	Oh et al. (2019)
Grapes (*Vitis vinifera* L.)	SSR	Riaz et al. (2018)
Plum (*Prunus* spp.)	ISSR (inter-simple sequence repeats)	Wu et al. (2018)
Sweet cherry (*Prunus avium* L.)	SSR	Patzak et al. (2019)
Almond (*Prunus dulcis* (Mill.) D.A Webb.)	SSR	Halasz et al. (2019)
Apple (*Malus ×domestica* Borkh.)	SSR	Syed (2019)
Pecan nut (*Carya* spp.)	SSR	Zhang et al. (2020)
European chestnut (*Castanea sativa* Mill.)	RAPD (random amplified polymorphic DNA)	Nunziata et al. (2020)
Walnut (*Juglans regia* L.)	ISSR	Houmanat et al. (2020)
Apricot (*Prunus* spp.)	SNP	Li et al. (2020)
Peach (*Prunus persica* L. Batsch.)	SNP	Mas-Gomez et al. (2021)

A similar study was conducted for estimating divergence in 30 sandy pear (*Pyrus pyrifolia* Nakai.) selections with 26 SSR markers. On the basis of SSR dendrogram data provided by Jan (2016), the selections were divided into two groups with four subgroups and one independent selection. The similarity coefficients ranged between 0.12 and 0.69, depicting a wide diversity due to the nature of genotypes and interspecific hybridization. A total of 48 cultivars and 54 wild accessions of Slovenian hazelnut (*Corylus avellana*) were evaluated for the genetic variability and subsequently the population structure using AFLP and SSR markers. These studies demonstrated higher levels of genetic diversity, with mean dissimilarity values of 0.50 and 0.60 for cultivars and wild accessions, respectively. There were 49 SSR markers and 11 AFLP primer combinations that produced 532 and 504 polymorphic fragments, respectively, showing high levels of genetic diversity. Additionally, these accessions were categorized for seven kernels and 10 nut attributes, with some wild accessions exhibiting breeding potential. In all, 49 SSRs were prominently linked with nut and kernel traits. Later studies by Öztürk et al. (2017) demonstrated the first application of association mapping in hazelnut and revealed that molecular markers are linked to significant qualitative features.

Genotyping by sequencing was adopted to determine the genetic diversity in *Actinidia* species, including the native *A. arguta* of Korea. As stated by Oh et al., later investigations cleared the path for kiwifruit breeding programs to generate cultivars employing *A. arguta* that are disease and cold stress tolerant and may be utilized as a base material for further breeding improvements (2018). A genetic diversity analysis of 1, 378 wild and cultivated grapevine accessions (*Vitis vinifera* L.) around Central Asia and the Mediterranean Basin was conducted employing 20 nuclear SSR markers in order to understand the possible events of domestication, gene flow, and adaptive introgression (Riaz et al., 2018). Using 14 ISSR primers, Wu et al. (2018) examined the genetic diversity and structure of 33 plum (*Prunus* spp.) cultivars grown in Southern China. They observed a total of 146 bands, 130 of which were found to be polymorphic. This indicates that there is frequent genetic exchange across closed subpopulations and existence of genetic diversity within the population.

By using molecular SSR markers and 110 amplified polymorphic markers for hierarchical cluster analysis of genetic variability, Patzak et al. (2019) assessed the genetic diversity within sweet cherry accessions of Czech genetic resources. The resulting dendrogram was in line with the ancestry and geobotanical traits of individual accessions, which were divided into five clusters. In order to assess genetic diversity metrics, characterize genetic differentiation, and investigate the mechanisms underlying the maintenance of population structure and genetic diversity in almond, 15 SSR loci were genotyped on 86 accessions of diverse ancestries (Halasz et al., 2019). Genetic divergence studies were examined in 17 clonal rootstocks (M9, MM106, MM111) of apple (*Malus ×domestica* Borkh.) using eight SSR markers, revealing that rootstocks were highly diverse as the SSR dendrogram data grouped rootstocks into two clusters with two-way similarity coefficients ranging from 0.00 to 0.32 (Syed, 2019).

The distribution of simple sequence repeat (SSR) motifs in two draft pecan genomes was examined in a study by Zhang et al. (2020). In pecan, PCR (polymerase chain reaction) amplification validated 66 SSR loci. In this work, it was discovered that 22 new development markers can be used to advance genetic research in the genus *Carya*. According to Nunziata et al. (2020), a group of cultivars were initially defined to assess the extent of genetic variation using random amplified polymorphic DNA (RAPD) markers for the purpose of varietal identification in European chestnut (*Castanea sativa* Mill.). Also, high-resolution melting (HRM) was employed for SNP mining inside 64 expressed sequence tags (EST) that involved all linkage groups and was validated by target resequencing. Genetic diversity estimation among 33 local Moroccan walnut (*Juglans regia* L.) trees which were compared with eight Bulgarian varieties belonging to an ex situ collection was studied employing ISSR markers, grown in two contrasted agroecosystems. In this study by using 13 primers, 120 ISSR markers (reproducible) were generated, demonstrating the high rate of polymorphism among genotypes with 7 to 13 bands and an average of nine bands per primer. According to these investigations, the overall polymorphism level (average) was 75.2%, with an average polymorphic informativeness content of 0.32 and a range of 0.212 to 0.370 (Houmanat et al., 2020). From these studies, it was concluded that ISSR markers were found to be more systematic in measuring the genetic diversity of walnut genotypes among two major areas in Morocco in comparison with foreign varieties as reported by Houmanat et al. (2020).

By using restriction site-associated DNA sequencing (RAD-seq) to sequence 168 apricot (*Prunus* spp.) accessions distributed in five ecological groups, Li et al. (2020) examined the genetic diversity and genetic relationships of these accessions. This study included 74 accessions of cultivated apricots (*Prunus armeniaca* L.) and 94 accessions of wild apricots (*Prunus armeniaca* L. and *Prunus sibirica* L.). This study provided authentic and relevant genomic resources to significantly promote apricot improvement and its effective utilization. Genome-wide diversity in peach germplasm in Spain has been explored by a new high-density (HD) Illumina peach SNP chip (9 + 9K). In this study, 9, 796 SNPs were used to genotype 90 peach accessions. There were 15 separate groups with genetically similar members found with the aid of identity-by-descent (IBD) estimate analysis. This study elucidated exchange of valuable germplasm among various regions of Spain for efficient management of the National Peach Germplasm Collection for preservation of genetic resources and benefit of impending genome-wide association studies (GWAS) of economically important fruit-related traits in peach as reported by Mas-Gomez et al. (2021). Although different DNA markers have been successfully used in estimation of genetic diversity as well as categorization of genetic material (Tiwari et al., 2013; Naeem et al., 2015; Wang et al., 2015), nowadays marker platforms particularly diversity array technology (DArT), as reported by Sanchez-Sevilla et al. (2015) for diversity analysis in octoploid cultivated strawberry (*Fragaria × ananassa*), are most commonly used for more efficient and reliable estimation.

## Gene tagging

When a gene is at close proximity to a known genetic marker, it is termed as “tagged.” Since the genetic markers operate as “signs” or “flags” rather than as the target genes themselves, they represent the genetic variation between certain organisms or species. Gene “tags” are genetic markers that are present physically next to genes (i.e., closely related to them). Due to their proximity to or “linking” to the genes governing the trait, these markers do not affect the phenotyping of the trait of interest by themselves (Collard et al., 2005). Molecular markers are being extensively used for breeding of modern temperate fruit crops for the selection of desired phenotypic traits. There are several previous studies demonstrating the applications of molecular markers for gene tagging in temperate fruit and nut crops (**Table 2**). Chaney et al. (2015) studied various traits in pecan (*Carya illinoinensis*). Genomic areas containing EST sequences, conserved gene sequences from various plant species, and simple sequence repetitions were amplified using PCR. This study offers a foundation for characterizing germplasm and locating molecular markers connected to features of practical significance.

**Table 2:** Traits of economic importance tagged with molecular markers in temperate fruit and nut crops.

Trait/gene	Fruit/nut crop	Markers/analysis	References
Nut characteristics, disease resistance and maturity	Pecan (*Carya illinoinensis* (Wangenh.) K.Koch)	CTAB method (cetyltrimethylammonium bromide)	Chaney et al. (2015)
Sex-linked polymorphism	Kiwifruit spp.(*Actinidia arguta*) and (*Actinidia kolomikta*)	GBS (genotyping-by-sequencing) analyses/SNP	Hale et al. (2018)
Apple flesh firmness (ACO and ACS)	Apple (*Malus × domestica* Borkh)	Md-ACS1 and Md-ACO1	Rasool (2018)
Sugar accumulation in apple	Apple (*Malus × domestica* Borkh.)	SSR and CAPS (cleaved amplified polymorphic sequence)	Zhen et al. (2018)
Almond blooming time	Almond (*Prunus dulcis* (Mill.) D.A Webb.)	RAPD and SSR	Rasouli et al. (2018)
Brachytic dwarfism (*Dw*)	Peach (*Prunus persica* L. Batsch.)	SNP	Cantin et al. (2018)
Self-incompatibility in Sweet cherry (*S*-locus)	Sweet cherry (*Prunus avium* L.)	PCR analyses	Patzak et al. (2019)
Fruit quality traits	Apricot (*Prunus armeniaca* L.)	qPCR (quantitative polymerase chain reaction) analyses	Garcia-Gomez et al. (2019)
Dwarfism (*PcDw* locus)	Pear (*Pyrus communis* L.)	qRT-PCR (quantitative reverse transcription polymerase chain reaction) analyses	Xiao et al. (2019)
Phenology, yield, and pellicle color	Persian walnut (*Juglans regia* L.)	Axiom™ 700K SNP array and GWAS (genome-wide association study)	Marrano et al. (2019)
Epistatic suppression of repeat fruiting	Strawberry (*Fragaria × ananassa* Duch.)	SSR	Lewers et al. (2019)
Cultivar improvement	Hazelnut (*Corylus avellana*)	SSR	Frary et al. (2019)
Phenology, fruit quality, and post-harvest quantitative parameters	Japanese plum (*Prunus salicina* Lindl.)	GLM (generalized linear model)/MQM (multiple-QTL mapping) analyses	Salazar et al. (2020)
Stem photosynthetic capability (SPC)	Wild almond (*Prunus arabica* (Olivier) Meikle)	SNPs	Brukental et al. (2021)

As reported by Hale et al. (2018), in two different species of kiwifruit, *viz*., *Actinidia arguta* and *Actinidia kolomikta*, sex-linked polymorphism was investigated *via* the genotyping-by-sequencing (GBS) method and from where independent populations were validated from both the species which were then converted into PCR markers (gel-based) for marker-assisted breeding programs. In this study, a 157-bp GBS amplicon marker for *Actinidia arguta* was created, and it displayed absolute sex linkage throughout the entire series of the kiwifruit germplasm. In a similar way, a 161-bp marker for *Actinidia kolomikta* was created with specific male allele primers pertaining to a 3-bp indel in a 270-bp GBS fragment, which displayed absolute sex linkage. The sex screening estimations of seedling populations with higher throughput now frequently use both of the markers, which were validated in separate populations.

Marker analysis has been done for fruit firmness by screening of 40 genotypes of *Malus × domestica* Borkh. (apple) maintained at the Division of Fruit Science SKUAST-Kashmir. In this study, the Md-ACO1 and Md-ACS1 markers were adopted and it was found that there are two allelic forms of Md-ACO1 and Md-ACS1, which are amplified in the forms Md-ACO1-1, Md-ACO1-2, and Md-ACS1-1 and each having three allelic combinations as ACS1-1/1 and ACS1-1/2, ACS1-2/2 and ACO1-1/1, and ACO1-1/2 and ACO1-/2/2, out of which ACS1-2/2 and ACO1-1/1 confirm high firmness with low ethylene production. Out of the 40 genotypes, for Md-ACS1, three were found to be homozygous ACS1-2/2 which include Gala Redlum, Gala Mast, and Fuji Zhen Aztec and were found to be highly firm. For Md-ACO1, three genotypes were found to be homozygous ACO1-1/1, including Red Velox, Shalimar Apple-1, and Oregon Spur which were found to be firm (Rasool, 2018). MdSWEET genes governing the sugar accumulation with high expression in *Malus × domestica* Borkh. were identified by Zhen et al. (2018), through development of gene-tagged SSR and CAPS (cleaved amplified polymorphism sequence) markers which revealed considerable phenotypic variation, thereby serving as an efficient tool in apple-breeding programs for genetic improvement of fruit sweetness.

To determine the time of flowering in *Prunus dulcis* Mill. D.A Webb. (almond), 140 RAPD primers, 87 nuclear SSR markers, and five chloroplast SSR markers were used. The results of this study directly indicated that the trait is quantitatively inherited. This study is also applicable to other *Prunus* species as mentioned by Rasouli et al. (2018). Cantin et al. (2018) detected one new allele for the gene *Dw* determining brachytic dwarfism in *Prunus persica* (peach). The gibberellin-insensitive dwarf 1 (G1D1) gene underwent a single-nucleotide polymorphism (SNP) mutation that was described in this study. In peach breeding programs, this study was used as a marker-assisted selection tool and undertaken to verify this marker in the F_2_ population of the cultivar “Nectavantop.” Using PCR molecular techniques, Patzak et al. (2020) evaluated the S-incompatibility locus in 153 accessions of sweet *Prunus avium* L. (cherry), identifying 13 distinct S-alleles in 29 S-locus combinations for 24 distinct incompatibility groups. The most prevalent S-alleles in this investigation were S1 (60 cultivars), S2 (34 cultivars), S3 (88 cultivars), S4 (54 cultivars), and S6 (28 cultivars).

In a study by Garcia-Gomez et al. (2019), *Prunus armeniaca* L. (apricot) was examined for eight important fruit quality traits, *viz*., fruit diameter, fruit weight, stone weight, blush color, skin ground color, firmness, acidity content, and soluble solid content. QTLs associated with these traits were identified, and the biological validity of these QTLs was assessed using qPCR gene expression analysis. Xiao et al. (2019) studied candidate genes governing dwarfism in pear revealed by comparative transcriptome analysis. This study offered a systemic perspective on the intricate regulatory networks governing the dwarf and standard phenotypes of the pear. The genetic regulation of Persian walnut (*Juglans regia* L.) phenology, yield, and pellicle color was uncovered by Marrano et al. (2019). In this study, the most recent Axiom™ *J. regia* 700K SNP array was used to create genetic profiles using phenotypic data. Furthermore, these research findings made a turning point in Persian walnut breeding as it moved from traditional to genome-assisted breeding.

By creating advanced markers (SSR) for the repeat fruiting region, Lewers et al. (2019) reported evidence of epistatic suppression of repeat fruiting in cultivated strawberries. This trait in strawberry is under the control of a dominant allele at a single locus, which has been formerly mapped by numerous research groups. It was found that repeat fruiting is suppressed by two more genes, among which one is dominant and the other one is recessive. The European hazelnut (*Corylus avellana*) was studied by Frary et al. (2019) by combining phenotypic data of kernel and nut with the corresponding genotypic data resulting from 406 SSR marker alleles with association mapping of quantitative trait loci for the respective traits. This analysis resulted in the identification of loci for cultivar improvement and revealed the effect of domestication and subsequent selection on kernel and nut traits. In this study, 78 loci were found, with the largest percentages for the parameters of the kernel (26%) and nut (24%) followed by other attributes like shell thickness (16%), quality (19%), and yield-related (15%) attributes. The loci regulating fruit quality, phenology, and post-harvest quantitative characteristics in *Prunus salicina* Lindl. (Japanese plum) were investigated, as found by Salazar et al. (2020). By using two genome association QTL analysis methodologies, general linear model (GLM)-based single marker–trait association and multiple QTL model analysis, 23 phenotypic traits were assessed over three harvest seasons to find the best linear unbiased predictors (MQM). According to these investigations, the most important QTLs in LG (linkage groups) were 4 and 2, which were related to the fruit weight and fruit developmental periods, respectively. In contrast, it was noted that small QTLs for fruit firmness evolution were validated in LG 4 and 5 using destructive and non-destructive approaches. In the existing and next breeding efforts for *Prunus salicina*, this study will offer useful information for MAS. *Prunus arabica* (Olivier) Meikle is a wild species of almond where there is assimilation of CO_2_ at greater levels by its green stem in the winter period on comparison with *Prunus dulcis* cv. Um el Fahem (U.E.F.) improving rates of carbohydrates in the dormant period. While unrevealing the inheritance pattern along with the mechanism for stem photosynthetic capability (SPC) in *Prunus arabica*, an F1 segregating population has been developed through crossing between *Prunus arabica* and *Prunus dulcis* cv. Um el Fahem (U.E.F.). The whole genome of both the parent plants has been sequenced, and with identification of 4, 887 informative SNPs for genotype assessment. There was the generation of a genetic map (robust) for *Prunus dulcis* cv. Um el Fahem (U.E.F.) and *Prunus arabica* involving (971 and 571 markers, respectively). Through mapping *via* QTLs (quantitative trait loci) and an association study (AS) with the phenotype for SPC, it was found that there were major QTLs [log of odd (LOD) = 20.8] upon chromosome number 7 and another minor (significant) upon chromosome number 1 (LOD = 3.9). This study investigated the physiology of the SPC trait in almond nut crop to breed varieties acquainted to winter temperature (Brukental et al., 2021). These studies indicate that the evolving gene-tagged markers are quite efficient to explore the functionality of genes and can be directly employed in temperate fruit and nut breeding programs in case of any association with horticultural traits of interest.

## Gene mapping

**Figure 1:**
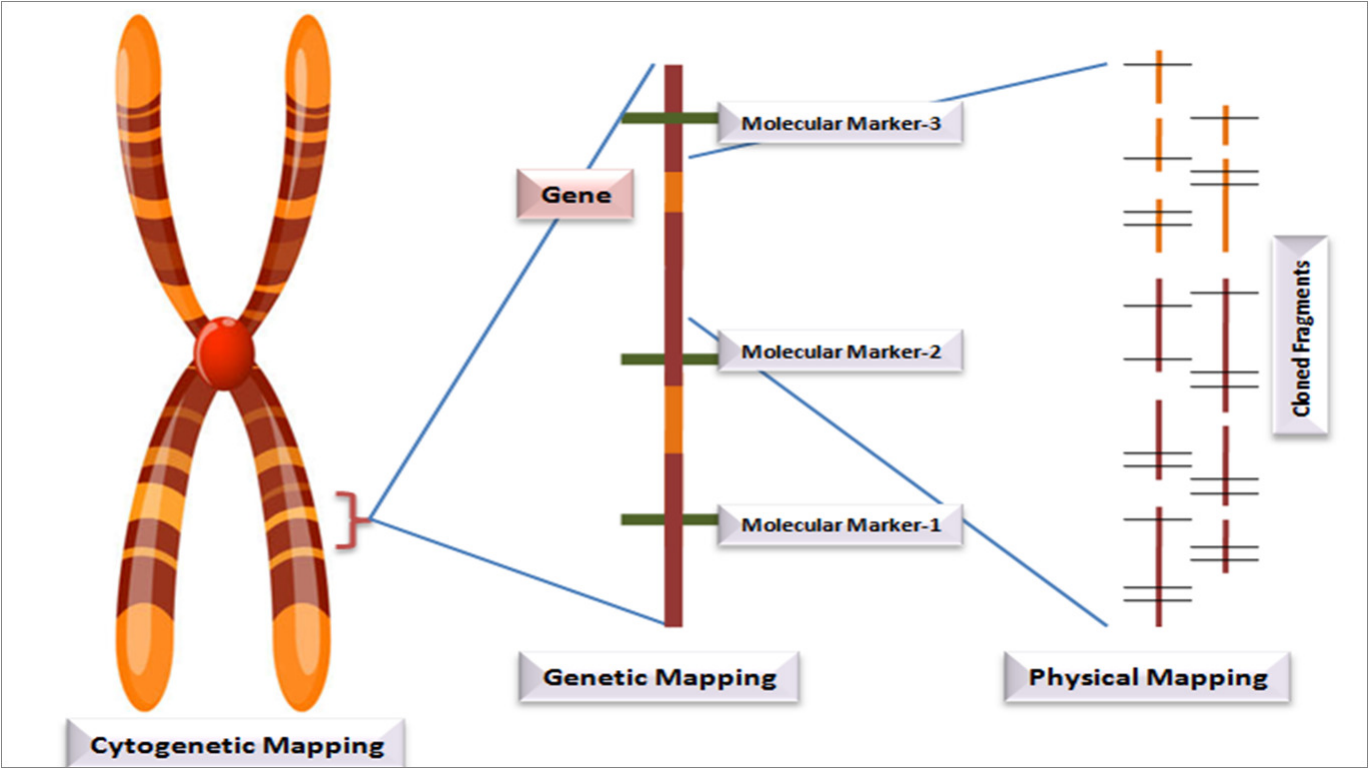
Large-scale gene mapping.

**Figure 2:**
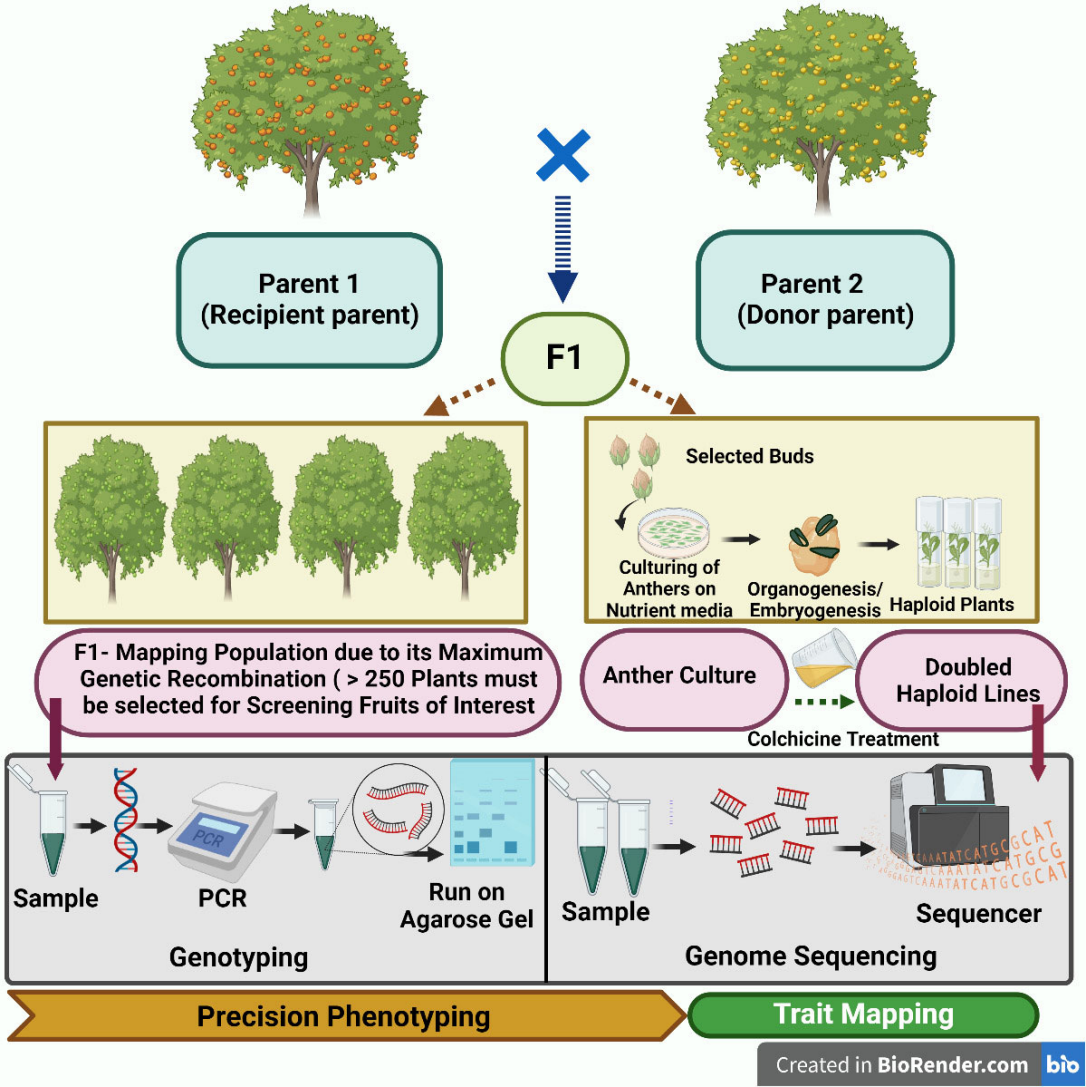
An outline of mapping of genes/QTL in fruit crops.

Another important subject of molecular marker utility is the preparation of molecular maps that help in identifying genomic regions controlling the horticultural important traits in temperate fruit crops. On the basis of markers of phenotypic nature, traditional linkage mapping allocates a specific location of genes for a desired chromosome. Based on cytogeny, there can be localization of genes for a specific band of chromosomes like association of the improved phenotype through deleting a desired band of chromosomes. Vast areas of a chromosome require the molecular marker approach for mapping in case of a specific type of special restriction endonucleases which check the specific sites as per intervals that are non-frequent. Into those fragments, subdivisions can be made and mapping can be done *via* endonucleases of traditional origin. Also, the contig type of maps locates desired genes with a specified fragment for precise gene mapping. The large-scale mapping of genes is shown in **Figure 1**. The principle behind gene mapping is the chromosomal recombination during meiosis, which results in the segregation of genes (Nadeem et al., 2018). Till date, QTL mapping and genome-wide association studies have successfully been implemented for mining several desirable alleles in a diverse range of crops and fruits species (Samantara et al., 2021). An overview of the QTL mapping is depicted in **Figure 2**. A vast range of linkage maps has been constructed in various temperate fruit crops employing the molecular marker approach (**Table 3**). Wang et al. (2015) created a mapping population from ‘Wanhongzhu’ and ‘Lapins’ and, moreover, constructed a linkage map in commercial sweet cherry along with QTL analysis for trunk diameter. This study used the SLAF sequencing technique (specific locus-amplified fragment) that included an SNP (single-nucleotide polymorphism) platform that produced 701 genotypic assays, together with 16 SSRs and the S (incompatibility) gene to prepare a map that covered 849.0 cM of distance involving eight LGs with a mean distance of 1.18 cM and had few gaps longer than 5 cM (centimorgan). This map demonstrated that four loci, corresponding to three distinct LGs (linkage groups), are responsible for controlling trunk diameter. This high-density map favors better-resolution identification of QTLs for horticultural important traits and speeds up improvement of sweet cherries. A genetic linkage map of *Castanea crenata* Sieb. Et Zucc. (Japanese chestnut) was prepared by Nishio et al. (2018) using two breeding populations, *viz*., ‘Kunimi’ and ‘709-034 (Kx709) and ‘Porotan’ and ‘Tsukuba-43’ (Px43). In this study, maps of four parents along with two integrated ones were constructed employing 443 SSRs and 554 SNPs. Out of which, Kx709 was the most saturated map involving 12 LGs, covering 668 and 1 cM, with an inter-distance of 0.8 cM. By using anchor SSRs, all the six maps were aligned to a Chinese chestnut consensus map. Eight agronomic traits were evaluated to identify molecular markers associated with them and at least one QTL was detected for each of the traits. With the help of this map, it was revealed that these mapping populations and their parents are essential material for Japanese chestnut breeding programs with these QTLs to be used for MAS to improve breeding programs. In the Japanese plum (*Prunus salicina* L.), Carrasco et al. (2018) created a linkage map that was intensely saturated utilizing GBS for SNP calling, the mapping population (*Angelenox aurora*), and the identification of 49, 826 SNPs while using the V2.1 peach genome as a reference. After rigorous filtering, 137 (*Angelenox aurora*) offspring revealed about 1, 441 SNPs of high quality that were mapped in eight linkage groups. An inter-distance of 0.96 cM was created utilizing 732 SNPs spanning 617 cM to create a consensus map. This map revealed a greater degree of collinearity and synteny between the genomes of peach and Japanese plum.

**Table 3:** Development of molecular maps in temperate fruit and nut crops.

Fruit/nut crop	Parents/population used	Marker type and number	Total map length (cM) and linkage groups (LG)	References
Sweet cherry (*Prunus avium* L.)	‘Wanhongzhu’ and ‘Lapins’	SNPs and 16 SSRs	849.0 cM 8 LG	Wang et al. (2015)
Japanese chestnut (*Castanea crenata* Sieb. Et Zucc.)	‘Kunimi’ and ‘709-034 (Kx709) ‘Porotan’ and ‘Tsukuba-43’(Px43)	554 SNPs and 443 SSRs	668.1 cM 12 LG	Nishio et al. (2018)
Japanese plum (*Prunus salicina* Lindl.)	Angelenox aurora	732 SNPs	617 cM 8 LG	Carrasco et al. (2018)
Walnut (*Juglans regia* L.)	Chandler × Idaho	2, 220 SNPs	1, 141.1 cM 16 LG	Aradhya et al. (2018)
Almond (*Prunus dulcis* (Mill.) D.A Webb.)	Gulcan-2 × Lauranne	168 SSR	47.8–84.6 cM 8 LG	Paizila et al. (2019)
Strawberry (*Fragaria × ananassa.*)	Maehyang × Albino	13, 181 SNPs	2581.57 cM 46 LG	Hossain et al. (2019)
Apricot (*Prunus armeniaca* L.)	‘Chuanzhihong’ and ‘Luotuohuang’	1, 991 polymorphic markers	886.25 cM 8 LG	Zhang et al. (2019)
Korean pears (Pyrus hybrid)	‘Whangkeumbae’ and ‘Minibae’	321 SNPs and 30 SSRs	1, 511.1 cM 17 LG	Han et al. (2019)
Apple (*Malus ×domestica* Borkh.)	‘ Fuji’ × ‘Red 3’	7, 630 SNPs	2, 270.21 cM 17 LG	Yang et al. (2020)
Grapes (*Vitis vinifera* L.)	Muscut Hamburg × Crimson Seedless F_1_ population	26, 039 SNPs	1463.38 cM 19 LG	Jiang et al. (2020)
Sweet cherry (*Prunus avium* L.)	‘Vic’ × ‘Cristobalina’ F_1_ population	910 SNPs (Vic) 789 SNPs (Cristobalina)	636.7 cM (Vic) 666.0 cM (Cristobalina)	Calle et al. (2021)

Aradhya et al. (2018) built a fine-scale genetic linkage map with 2, 220 SNPs in 16 linkage groups with a mapping population, spanning 1, 141.1 cM, deciphering genomic regions governing economic traits *viz*., yield, lateral fruitfulness, harvest date, nut weight shell thickness, and kernel fill in walnut (*Juglans regia*). At LG 1, two to three pleiotropic QTLs were in charge of controlling the thickness of the shell, nut weight, and kernel fullness. QTLs showed a negligible impact on such trait expressions, as seen by their tiny positive cumulative effects for shell thickness, harvest date, and nut weight and minor negative cumulative impacts for kernel fill. Using the Gulcan-2 × Lauranne mapping population, an SSR-based linkage map for almonds was created. The consensus map has 168 markers spread across eight LGs. According to this study, there were 13 mapped markers (LG 6) and 31 markers (LG 2), and the length of the LGs ranged between 47.8 cm (LG 6) and 84.6 cm (LG 1), with a mean distance of 3.1 cm. This study came to the conclusion that this map served as a foundation for the development of markers associated with critical almond features after the completion of the phenotypic data, as reported by Paizila et al. (2019).

Using the ddRAD (double digest restriction-associated DNA) sequencing approach, Hossain et al. (2019) was able to identify 13, 181 SNPs for the development of a high-density linkage map and QTL mapping for runner production in *Fragaria* × *ananassa* (octaploid strawberry). Mendelian segregation was detected in 3, 051 SNPs in F1, among which 1, 268 were linked to 46 linkage groups covering 2, 581.57 cM with a 2.22-cM intergenic distance. In addition, seven QTLs were found for runner production, the LOD values of which ranged between 3.5 and 7.24 and phenotypic variances of 22–38%. From this research, it was concluded that both vegetative and reproductive behaviors of strawberry could be determined and can serve as a tool for breeding targets regarding flowering and runner production. Zhang et al. (2019) crossed two Chinese cultivars, “Chuanzhihong” and “Luotuohuang, “ along with a recently built reduced representation library, to create a high-density linkage map and QTL analysis for pistil abortion in *Prunus armeniaca* L. (apricot). A total of 12, 451 polymorphic markers were created for this map using resequencing data; the final map was composed of eight linkage groups with 1991 markers encompassing 886.25 cM, with a mean distance of 0.46 cM. This is the most detailed genetic map of the apricot currently available. This map was used in this study to identify the QTL responsible for apricot pistil abortion by analyzing the central regions of LG 5 (51.005–59.4 cM) and LG 6 (72.884–76.562 cM), which contained nine SLAF markers that were strongly associated with pistil abortion. It was determined by this analysis to be a very valuable connection map. This study deciphered that it is a quite valuable linkage map for identifying QTLs related to different agronomic traits in apricot and the well-organized markers are useful for molecular breeding of apricot.

Han et al. (2019) created an integrated linkage map of Korean pears (*Pyrus hybrid*) as per the pseudo-chromosomes of the reference genome of the Chinese pear known as “Dangshansuli” (*Pyrus bretschneideri*), using GBS-based SNPs and SSRs with consensus maps for “Whangkeumbae” and “Minibae” that contains 321 SNP and 30 SSR markers with 1511.1 cm with an average genetic distance of 4.3 cm. This consensus map was compared to other apple and pear maps using 30 SSR markers in total. According to the results of this investigation, SSRs coming from pear and apple maps exhibit strong synteny. With the help of pseudo-chromosomes and SNPs, 17 linkage groups of this genus’ physical length were revealed, and genome-based regions that were discovered by QTL analysis were annotated. The genotyping by sequencing-based SNPs integrated with SSRs provide a description of the genome structure of the Korean pear resources serving as a reference map. A high-density linkage map was constructed in apple (*Malus × domestica* Borkh.) employing the ‘Fuji’ × ‘Red 3’ population with homozygous alleles R1R1 and R6R6, respectively, by Yang et al. (2020). The linkage map contains 7, 630 SNPs that distinguish between “Fuji” and “Red 3, “ of which 3, 903 and 3, 925 were mapped into the linkage groups of the parents, respectively, the male and the female. The number of “Fuji” SNP markers for the female parent linkage group ranged from 108 to 366, with a mean of 231. The distance between all linkage groups ranged between 70.17 and 168.23 cM, with a mean value of 109.14 cM. Each linkage group’s SNP count ranged from 58 to 434 for the male father “Red 3, “ with an average of 230. Each linkage group’s distance ranged between 72.11 and 227.10 cM, with a mean distance of 140.26 cM. With 17 linkage groups and a mean density of 0.30 cM per marker, the consensus map covered 2, 270.21 cM. With 0.19 cM per marker, LG10 exhibited the lowest heterozygosity, whereas LG17 displayed a high level of heterozygosity at 0.73 cM per marker, according to this map. The SNP density of other 15 LGs ranged between 0.21 and 0.48 cM.

In order to discover loci determining the firmness of the berry in grapes, a high-density genetic map with 19 linkage groups, 1, 662 bin markers including 26, 039 SNPs, spanning a total distance of 1, 463.38 cM, and an inter-marker distance of 0.88 cM was generated (*Vitis vinifera* L.). Four potential genes related to abscisic acid (ABA), endoglucanase, and transcription factors were linked in this study to berry hardness. Using qRT-PCR research, it was discovered that Muscat Hamburg had higher levels of the endoglucanase 3 gene and abscisic-aldehyde oxidase-like gene expression at the veraison stage than Crimson Seedless, which was consistent with the parent berry firmness. The above two genes were found to be the candidate genes determining berry firmness in grapes as reported by Jiang et al. (2020). In the sweet cherry (*Prunus avium* L.) ‘Vic’ × ‘Cristobalina’ F_1_ population, QTL mapping of phenolic substances and that of cherry fruit color was done by Calle et al. (2021) using 6 + 9K SNPs, 910 SNPs (Vic), and 789 SNPs (Cristobalina) array genetic map with a map distance of 636.7 cM (Vic) and 666.0 cM (Cristobalina). These studies reflect that the genetic linkage maps play a vital role to explore the genetic control of important traits and serve as a DNA-based diagnostic tool in future for marker-aided breeding techniques.

## Abiotic and biotic stress tolerance

Molecular markers have made it possible to impart abiotic and biotic stress tolerance in temperate fruit and nut crops by tapping resistant genes from their wild relatives and incorporating them into commercial cultivated varieties, thereby reducing the yield losses due to various stresses (**Table 4**). Zhu et al. (2015) built a high-density genetic map of anthracnose resistance in walnut according to specific AFLP markers (total 153, 820), out of which 2, 577 markers were employed to build genetic linkage maps (448 for male, 2, 395 into 16 LGs for female map, and 2, 577 for integrated map). By considering the range of all LGs, the marker coverage for the integrated map was 2, 457.82 cM, with an average interval of 0.95 cM. In addition, 5, 043 SNP loci correspond to two SNP loci per SLAF marker. In this study, for identifying anthracnose resistance, QTLs were detected ranging from 165.51 to 176.33 cM on LG 14. The phenotypic variation in this study varied from 16.2 to 19.9% with LOD scores (logarithm of odds) (3.22 to 4.04). This study is a base for aiding molecular marker-assisted breeding for identification of walnut resistance gene. A study was described by Montanari et al. (2016) who detected a QTL on LG 2 using a previously conducted genetic map published in 2013 with SSR and SNP markers for the segregating population of PEAR3x ‘Moonglow’. This QTL corresponds with the QTL for fire blight disease resistance earlier found in ‘Harrow Sweet’ (Le Roux et al., 2012), additionally giving an insight that these two favorable alleles were not identical by descent. In this study, few little effect QTLs were identified from susceptible parent PEAR3. This study proposed that the SNP and SSR markers were linked to the higher effect QTL on LG2 candidates for MAB for resistance of pear fire blight disease. Lenz and Dai (2017) mapped X-disease phytoplasma resistance in *Prunus virginiana*, employing chokecherry consensus map “Cho” containing 16 LGs covering 2, 172 cM with average marker density of 3.97 cM. Also, three QTLs were associated with this trait, accounting for about 45.9% of phenotypic variation. This study deciphered a framework for genomics, molecular genetics, and further breeding aspects for X-disease resistance in Chokecherry and other *Prunus* species. Ink disease resistance in European chestnut was imparted by Santos et al. (2017) through an approach involving construction of a genetic linkage map from *Castanea sativa* × *Castanea crenata* for detection of QTL for this trait. In this study, chestnut populations were genotyped with 452 SNPs and microsatellites derived from available chestnut transcriptomes. Non-parametric approaches and composite interval mapping identified two QTLs in two LGs, E and K. The presence of QTL in LG E is in accordance with a previous study by Santos et al. (2015) in American × Chinese chestnut populations, depicting the presence of a defense system throughout the species. From the above research, it was concluded that genomic resources of *Castanea* genus act as a tool for future breeding aspects.

**Table 4:** DNA markers for abiotic and biotic stress tolerance in temperate fruit and nut crops.

Trait/resistance	Fruit crop/parents	Markers/genes/technique	References
Anthracnose	Walnut (*Juglans regia* L.)	SLAF (specific locus-amplified fragment)/SNPs	Zhu et al. (2015)
Fire blight	PEAR3 (PremP003, *P.* × *bretschneideri* × *P. communis*) × ‘Moonglow’(*P. communis*)	QTL	Montanari et al. (2016)
X-Disease	Chokecherry (*Prunus virginiana*)	QTL	Lenz and Dai (2017)
Ink disease	*Castanea sativa* × *Castanea crenata*	Microsatellite/SNPs	Santos et al. (2017)
Powdery mildew	Strawberry (*Fragaria × ananassa.*)	QTL	Cockerton et al. (2018)
Apple scab	Apple (*Malus × domestica* Borkh.)	AL07 and AM19 (SSR)	Rasool (2018)
Brown rot	Almond ‘Texas’ × Peach ‘Earlygold’ population	QTL	Baro-Montel et al. (2019)
Downy mildew	Grapes (*Vitis vinifera* L.)	SSR/RT-qPCR (quantitative reverse transcription polymerase chain reaction)	Zhao et al. (2019)
Sharka disease	Apricot (*Prunus armeniaca* L.)	SNP	Mori et al. (2019)
Wilt	Kiwifruit (*Actinidia* spp.)	SSR	Oliveira et al. (2020)
Powdery mildew	Peach (*Prunus persica* L. Batsch.)	Vr3/*Prupe2G111700*/*Prupe2G112800*	Marimon et al. (2020)
Black spot	Japanese pear (*Pyrus pyrifolia* (Burm.) Nak.)	SSR	Terakami et al. (2021)
Abiotic stress	Apple (*Malus* × *domestica* Borkh.)	qRT-PCR/MdDREB2A transcription factor	Lian et al. (2021)
Codling moth *Cydia pomonella* (Linnaeus) (Lepidoptera: Tortricidae)	Sweet cherry *(Prunus avium* (L.))	PCR/(COI) gene	Yokomi et al., 2021)

For regulating abiotic stresses, viz., drought, salinity, and stresses due to ABA, in apple (*Malus ×domestica* Borkh.), the MdDREB2A transcription factor from the AP2/ERF family has been isolated from cultivar ‘Royal Gala’. By using quantitative reverse transcription polymerase chain reaction (qRT-PCR), it was found that the length of the open reading frame is 1, 197 bp encoding a 398-long amino acid chain protein with a molecular weight of 43.8 kD (Lian et al., 2021).

The fruit export can be easily facilitated with implementation of advanced molecular techniques to improvise the pest diagnostics in a faster way *via* polymerase chain reaction (PCR) and latest techniques of sequencing. The larvae of codling moth *Cydia pomonella* (Linnaeus) (Lepidoptera: Tortricidae) were found in a pack house of cherry *Prunus avium* (L.) being differentiated *via* PCR compared to various other internal fruit moth larvae, *viz*., the oriental fruit moth, *Grapholita molesta* (Busck) (Lepidoptera: Tortricidae); lesser appleworm, *G. prunivora* (Walsh) (Lepidoptera: Tortricidae); cherry fruitworm, *G. packardi* (Zeller) (Lepidoptera: Tortricidae); and filbertworm, *Cydia latiferreana* (Walsingham) (Lepidoptera: Tortricidae). Also, there is conformity of identifying pests *via* amplicon sequencing (301 bp) part of the (COI) gene obtained *via* PCR in the DNA of suspected moth through comparison with other (COI) gene sequences from the rest of the internal fruit borers in fruits of the pome group. This study was conducted for meeting phytosanitary aspects to export fruits globally (Yokomi et al., 2021).

Resistance against powdery mildew was identified by Cockerton et al. (2018) using genotypes from two phenotyped bi-parental mapping populations, “Emily” × “Fenella” and “Redgauntlet” × “Hapil, “ to present quantitative trait loci in *Fragaria × ananassa* (strawberry). Multiple QTLs were found in this study, and six of them demonstrated a level of persistent resistance across different phenotyping events. It was examined whether found QTL showed close linkage to associated genes for resistance in the larger germplasm after the identified QTL was subjected to screening across an evaluation set. This study showed that quantitative resistance develops across the strawberry germplasm *via* several predominantly additive sources. An investigation was carried out to document and characterize the apple germplasm comprising 40 apple cultivars in which molecular characterization was performed for scab resistance using *AL07* (codominant primer) and *AM19* (dominant primer), and the results obtained with primers confirmed the presence and absence of the *Vf* gene. It was also found that *AL07* (codominant primer) are helpful to discriminate homozygous from heterozygous plants for the *Vf* gene as analyzed by Rasool (2018). As reported by Baro-Montel et al. (2019), resistance to brown rot disease caused by *Monilinia* spp. in stone fruits as explored in an interspecific almond ‘Texas’ × peach ‘Earlygold’ population (named T1E) was identified. This study revealed that ‘Texas’ is a resistant source for brown rot disease and many quantitative trait loci were detected in various linkage groups, but two proximal QTLs were identified in G4, accounting for 11.3–16.2% of phenotypic variation. Hence, a MAS-based strategy is needed to incorporate this trait into the susceptible parent peach with other desirable traits for sustainable crop production.

The variations in the rates of downy mildew resistance of various Chinese wild grapevine were identified by Zhao et al. in 2019. In this study, *in vitro* leaf disc testing was used to assess the level of downy mildew resistance, and RT-qPCR (quantitative reverse transcription polymerase chain reaction) was used to determine the expression pattern of various important genes following *Plasmopara viticola* inoculation. Using 30 pairs of SSR markers, 120 grapevine germplasm resources were chosen, showing a disease index range of 0.00 to 78.70. The RGA9 marker was a standard marker that, among other markers, expressed the highest phenotypic variation of 80.83% that may be used in the generation of disease resistance, providing the basis for widespread as well as rapid discovery of downy mildew disease resistance in many organisms. Mori et al. (2019) reported that resistance to sharka disease in apricot was identified by comparing phase-constructed susceptible and resistant haplophytes of ‘Lito’ and Chromosome 1 along with assessment of candidate genes. A pseudo-test cross-mating design with 231 individuals was followed to discover the fact that the ‘Lito’ genotype had heterozygosity for the resistance that has been used to map a significant QTL on LG 1. Additionally, this analysis included 19 SNP markers that covered 236 kb of chromosome 1 in total. A BAC (bacterial artificial chromosome) library for the ‘Lito’ cultivar was created, and markers for the area were used to screen it. Positive BAC clones were then sequenced. The whole genome of the given cultivar was sequenced for refinement for a high coverage using PacBio technology, deciphering a detailed analysis of the genes in the QTL region. This cultivar drastically distinguished structural variants between two haplophytic regions and also predicted specific allelic expression leading to mining of the PPVres locus. Therefore, a valuable prediction method has been developed to study the substitute transcription and regulatory mechanisms underpinning PPV resistance.

Oliveira et al. (2020) conducted a study for resistance to *Ceratocystis* wilt in kiwifruit cultivars, employing 46 isolates with 14 SSR markers for genotyping. Among 14 markers, 13 were polymorphic and 26 genotypes were identified. On susceptible cultivar, 14 prominent genotypes were tested for identification of aggressive ones, eventually by inoculating an equal mixture of five of the most aggressive ones for evaluating seven resistant types, *viz*., Red Arguta, Green Arguta, Chieftain, Allison, Hayward, Monty, and Tomuri, which could be used as rootstocks for future programs. The powdery mildew-resistant (Vr3) gene in peaches was mapped to a genome area of 270 kb containing 27 identified genes to get molecular markers strongly connected to this gene. The results of this study’s analysis revealed that there was a difference in the expression of a gene known as Disease Resistance Protein RGA2 (Prupe2G111700) or Eceriferum 1 protein resulting in biosynthesis of epicuticular wax (Prupe2G112800) between resistant and susceptible individuals. Only Prupe2G111700 contains a variant that has an adverse impact on the protein encoded, which showed overexpression in both homozygous and heterozygous individuals. With the help of this study, molecular markers tightly linking and flanking the *Vr3* gene could be identified and validated as reported by Marimon et al. (2020). Terakami et al. (2021) developed the SSR marker set, *viz*., Mdo.chr11.27 and Mdo.chr11.34, for efficient selection for black spot disease resistance in pear breeding by performing inoculation tests and genotyping with 207 pear cultivars. In addition to this, with the molecular marker approach, pyramiding multiple resistant genes into the host plant with accelerated breeding programs provide durable and broad-spectrum resistance for further research in temperate fruit and nut breeding programs.

## Genome sequencing

The ultimate goal of genomics is sequencing of the whole genome and understanding of the function of each gene. For both functional genomics and breeding programs in fruit crops, the publication of a dozen genomes represents a milestone. This review provides some insights regarding various genome sequencing projects in temperate fruit and nut crops employing the molecular marker approach (**Table 5**). Along with high-throughput sequencing technology, rapid advances have revolutionized the manner and scale of genomics in temperate fruit and nut crops. Employing shotgun sequencing of a nearly homozygous genotype (cv.PN40024) which was originally derived from Pinot Noir by a French-Italian public consortium, the first 8X version of the *Vitis vinifera* genome sequence was obtained and was the first sequenced fruit crop (Jaillon et al., 2007). The presence of the grape genome sequence with a haploid chromosome number of 19, containing 490 Mb with 30, 000 protein-coding genes, is causing a rapid increase in *Vitis* genetic research by providing tools for genetic improvement to grow well with stresses like diseases and pests while maintaining specific fruit composition and a new research framework, which was confirmed by Martinez-Zapater et al. (2010).

**Table 5:** Status of sequencing projects in temperate fruit and nut crops.

Species	Common name	Genome size (Mb)	References
*Vitis vinifera*	Grapes	490	Martinez-Zapater et al. (2010)
*Malus × domestica* Borkh.	Apple	742	Velasco et al. (2010)
*Prunus persica* L. Batsch.	Peach	230	Arus et al. (2012)
*Fragaria × ananassa*	Strawberry	813.4	Hirakawa et al. (2014)
*Pyrus communis* L. ‘Bartlett’	European pear	577.3	Chagné et al. (2014)
*Juglans regia* L.	Walnut	667	Martinez-Garcia et al. (2016)
*Prunus dulcis* cv. Texas	Almond	238	Alioto et al., 2020
*Prunus armeniaca* L.	Apricot	221.9	Jiang et al. (2019)
*Actinidia chinensis*	Kiwifruit	653	Wu et al. (2019)
*Prunus fruticosa* Pall.	Ground cherry	366	Wohner et al. (2021)

A significant scientific milestone was reached for *Malus × domestica* Borkh. variety Golden Delicious in 2010 with the publishing of the first drought whole-genome sequence (WGS), which had a genome size of 742 Mb (Velasco et al., 2010). As the first reference for sequencing data, precise mapping, gene discovery, variation finding, and tool creation, this WGS, v1.0, proved useful. The reference genome for apples is now GDDH13v1.1, a fresh, high-quality whole-genome sequence for apple that was published in 2017. These whole-genome sequences of apple have a significant effect on understanding the biological functioning of apple, its trait physiology, and subsequently its inheritance, which find practical applications for enhancing this high-value crop, as stated by Peace et al. (2019), with immense potential for crop improvement. The genome sequencing of *Prunus persica* L. Batsch (peach) has just lately been made accessible to the scientists and researchers. With its short juvenile period (2–4 years) and self-mating design, the peach’s short (230 Mbp), simple, diploid distribution on eight pairs of chromosomes provide a great way to build a robust sequence of its entire genome, which improves the way for the rapid development of genomics in peaches (Arus et al., 2012). Guo et al. (2020) gave an integrated structural map of the peach genome with 202, 274 SVs (structural variations) which influence 2, 268 genes. Using genome-wide association studies, 26 horticultural traits with the help of those SVs detected various candidate variants, *viz*., 9-bp insertion in *Prupe.4G186800* (encoding NAC transcription factor) for early bearing and 487-bp deletion with the *PpMYB10.1* promoter for flesh color around the stone. In addition, 1.67 MB inversion relates to fruit shape and a gene adjacent to inversion breakpoint, *PpOFP1*, relates to flat shape type.

*Fragaria vesca* (woodland strawberry; 2n = 2x = 14), a herbaceous perennial with a genomic size of 240 MB, is highly specific to genetic transformation and shares a large sequence identity with *Fragaria × ananassa* (the cultivated strawberry). Through gene prediction modeling which is supported by transcriptome mapping, almost 34, 809 genes have been identified. These genes are valuable to critical horticultural traits like nutritional values, flavor, and flowering time. The strawberry sequence is diploid, whereas other rosids’ massive genomic duplications are absent (Shulaev et al., 2011). The assembly and annotation of the *Fragaria ×ananassa* (octoploid strawberry) genome have been completed in cultivar “Camarosa, “ with an average genome size of 813.4 Mb (Hirakawa et al., 2014). A high-quality reference genome is required for the octaploid strawberry in order to properly utilize it as a model system for investigating allopolyploidy and to provide a platform for discovering physiologically and horticulturally significant genes and using genome enabled breeding techniques (Collard and Mackill, 2008).

Using second-generation sequencing technology, Chagné et al. (2014) presented a dwarf assembly of the *Pyrus communis* (European pear) ‘Bartlett’ genome from single-end, 2- and 7-kb insert paired end reads using Newbler (v 2.7). This assembly included 142, 083 scaffolds longer than 499 bases, with a maximum scaffold length of 1.2 Mb, spanning 577.3 Mb, and its re-sequencing of the samples “Old Home” and “Louise Bonne de Jersey” allowed the detection of 8, 29, 823 potential SNPs. About 2, 279 genetically mapped SNPs anchor 171 Mb of the assembled genome. This study revealed that the draft assembly ‘Bartlett’ v 1.0 is an incredible tool for detecting important horticultural traits in Pyrus enabling marker-assisted and genomic selection for speeding pear breeding progress. To discover target genes and additional unknown genes, in Persian walnut (*Juglans regia* L.), the genome sequence was obtained from cultivar ‘Chandler’. Using two methods, viz., SOAPdenovo2 and MaSuRCA, a genome of the size 667 Mbp was congregated having an N50 scaffold size of 464, 955 bp (according to the genome size of 606 Mbp), 37% GC content, and 221, 640 contigs. The walnut genome sequence provides information to boost the breeding and favor the complex traits in genetic dissection as reported by Martinez-Garcia et al. (2016). In a recent study by Marrano et al. (2020), Chandler v2.0 (the new chromosome level assembly of the reference genome for walnut) was obtained employing combination of Oxford Nanopore long-read sequencing with Hi-C (chromosome conformation capture) technology. In comparison with the former reference genome, the new assembly possesses a 84.4-fold increase in N50 size and assemblage of 16 chromosomal pseudomolecules, accounting for 95% of the total length. With this Chandler v2.0, walnut biology can be better explored for future breeding aspects.

Alioto et al. (2020) attempted the genome sequencing of Texas, a highly heterozygous cultivar of almond (*Prunus dulcis*). From about 238-Mb estimated size of the almond genome, genome assembly of 227.6 Mb was obtained, from which 91% is clamped to eight pseudomolecules leading to its haploid chromosome constituent, elucidating about 27, 969 coding genes (protein) and 6, 747 non-coding transcripts. According to a phylogenomic analysis of 16 genomes of other nearby and far-off species, the almond and peach diverged roughly 5.88 million years before. With 20 nucleotide substitutions per kilobase, these two genomes exhibit remarkable sequence conservation and strong collinearity. Transposable elements (TE) have evolved into a variety of presence/absence variants between peach and almond, depicting a recent history of the movement of TE looks markedly between them, suggesting that TE share an important role in the history and diversification of peach and almond. This study revealed that TE have evolved into a variety of presence/absence variants between almond and peach. A high-quality reference genome has been elucidated by Jiang et al. (2019), using long-read single-molecule sequencing with 40.46 Gb, corresponding with a genetic map for chromosome scaffolding. The assembled genome covers 221.9 Mb, with a contig NG50 size of 1.02 Mb. In over eight chromosomes, scaffolds spanning 92.88% of the assembled genome were anchored. This study concluded that this highly contiguous reference genome of *Prunus armeniaca* will elucidate Rosaceae family evolution for future breeding endeavors by identifying agronomic traits of interest for further improvement. Based on PacBio long reads and Hi-C data, Wu et al. (2019) built a revised chromosome-level reference genome of *A. chinensis* (v3.0), which is 653 Mb long and contains 0.76% heterozygosity. At least 43% of the sequences in this genome repeat, and the most prevalent long terminal repeats, which make up 23.38% of the unique genome, have also been found.

Wohner et al. (2021) drafted chromosome-level genome assembly of tetraploid ground cherry (*Prunus fruticosa* Pall.) with 366 Mb comprising eight chromosomes to pave a way for breeding, molecular, and evolutionary studies in *Prunus*. With this research, it was suggested that molecular assessment of diverse genetic traits can be done for further breeding and evolutionary studies. In addition to this, advances are required to upgrade the basic nucleotide sequences in the genome of different temperate fruit crops as summarized by Peace et al. (2019), in apple with a view to improve the reference WGS (whole-genome sequencing) genome ensuring its accuracy and completeness so as to mark all the suspicious regions identified while comparing the WGS with high-quality genetic linkage maps. Also, advances in genome sequencing are required by devising a gene atlas of different temperate fruit and nut crops to describe the functions and relationships of different genes to explore the role of various putative protein-coding genes which are unknown.

## Omics approach in temperate fruits and nuts

Over the recent decades, the advent of several omics approaches has proven to be highly beneficial against different biotic and abiotic stress responses by developing novel crop species through modification of the genetic and molecular pathways *via* alteration of DNAs, transcripts, proteins, metabolites, and mineral nutrient levels (Muthamilarasan et al., 2019). The analysis of transcriptome, metabolome, and proteome profiles promotes insights on several functional roles of different genes. It also assists in identification and quantification of transcripts, non-coding RNAs, and pathways that are concerned with fruit traits such as sugar metabolism, their developmental and ripening process, shelf life, quality characteristics, and resistance mechanism under adverse physiological and environmental stresses (Zheng et al., 2021). Overall, the integrated omics approaches, *viz*., genomics, transcriptomics, metabolomics, and proteomics, could elucidate precise genomic information and may offer further opportunities for their genetic alteration *via* gene engineering tools like CRISPR (clustered regularly interspaced short palindromic repeats) and RNAi (RNA interference), in terms of better fruit quality and production through accelerating the long breeding generations (Mathiazhagan et al., 2021). Currently, GAB (genomics-assisted breeding) has tremendous utilization in terms of addressing better quality, yield, and several biotic and abiotic stress (drought and salt) resistances (in a wide range of crop species; Ganie et al., 2021; Wani et al., 2022). Also, several applications of omics platforms have been implemented in the way of different fruit improvements. For instance, a comparative transcriptomics study between unripe and ripe berries of grapevine demonstrated several ripening-linked secondary metabolisms, viz., anthocyanins (Wu et al., 2014), proanthocyanidins (Carrier et al., 2013), and volatile compounds (Cramer et al., 2014). A transcriptome analysis of Chinese pear showed high variation of gene expressions during the developmental phase of tissues and fruits (Xie et al., 2013). In strawberry, a multiomics approach revealed the association of different genes regulating volatile organic compounds behind the flavor characteristics of its berries (Fan et al., 2022). A comparative transcriptome analysis among several apple verities exhibited 34 genes regulating the anthocyanin contents which are associated with preferred appearance of apples (El-Sharkawy et al., 2015). Zaini et al. (2020) conducted a deep comparative proteomics study in kernel pellicle tissues of two elite walnut cultivars and detected proteins that are concerned with secondary metabolism governing the pigmentation leading to nut qualities. In avocado, an integrated proteomics and metabolomics study indicated the induction of ripening homogeneity through heat treatment that boosts the sugar soluble accumulation, *viz*., sucrose and galactose and other stress-related enzymes (Gavicho Uarrota et al., 2019). Similarly, another study has been carried out by combining transcriptomics with network analysis to determine the control mechanisms concerned with acid accumulation and citrate in sweet orange varieties that contributes to their fruit quality and tastes (Huang et al., 2016). These omics approaches have also played crucial roles in deciphering different gene regulations behind abiotic and biotic stress responses. For example, a metabolomics analysis in peach determined the association of different metabolites, *viz*., mannitol, galactinol, and raffinose, particularly higher raffinose accumulation under freezing stress conditions (Brizzolara et al., 2018). In case of biotic stress, a comparative proteomics study in resistant and susceptible apple leaves affected with *Alternaria alternate* disease demonstrated a major involvement of 43 differentially expressed proteins behind both pathogenesis and defense mechanism (Zhang et al., 2015). Another study through a quantitative proteomics analysis revealed up- and downregulation of several differential accumulated proteins that are associated behind gray mold disease of kiwifruits (Liu et al., 2018).

## Conclusions and future prospects

The temperate fruit and nut crops being perennial in nature undergoes several difficulties in terms of their genetic improvement through classical methods of breeding, which involves long-term deliberate crossing of closely and distantly related individuals to develop desired cultivars and varieties. Therefore, the advance molecular breeding approach since the last three decades have been widely adapted and assisted in revolutionizing the fruit crop improvements. The current advances in gene sequencing technologies have facilitated deep insights on genes regulating desirable fruit qualities, their ripening, and aroma development mechanisms, including biotic and abiotic stress responses. However, a systemic method in using sequence information on the complex pathways of fruit crop development is still less frequently utilized. Hence, an integrated systems biology approach through implementation of the multiomics approach which can generate functionally validated genomic knowledge is required for fruit improvement programs. The genome-wide association study and genomic selection approaches can assist in association of multiple traits and eliminate the requirement for developing mapping population, thus shortening the duration and space necessary for fruit orchard maintenance. Next, the cutting-edge technologies, *viz*., CRISPR-mediated genome editing, can be a beneficial asset in the way of transgene-free precise genetic modification that facilitate no genetic footprints and no off-targets in developing novel and resistant qualitative fruit and nut varieties. Hence, for improvement in commercial temperate fruit and nut crops, the integration of various biotechnological tools, *viz*., high-throughput molecular markers along with the CRISPR gene editing systems can accelerate the pace of precision breeding programs in future, opening greater avenues for research and sustainability to farmers and consumers.

## Author contributions

All authors listed have made a substantial, direct, and intellectual contribution to the work, and approved it for publication.

## References

[B1] Alioto T. Alexiou K. G. Bardil A. Barteri F. Castanera R. Cruz F.. (2020). Transposons played a major role in the diversification between the closely related almond and peach genomes:results from the almond genome sequence. Plant J. 101 (2), 455–472. doi: 10.1111/tpj.14538 31529539 PMC7004133

[B2] Al-Samarai F. R. Al-Kazaz A. A. (2015). Molecular markers:an introduction and applications. Eur. J. Mol. Biotechnol. 9 (3), 118–130. doi: 10.13187/ejmb.2015.9.118

[B3] Aradhya M. K. Velasco D. Wang J. Ramasamy R. You F. M. Lesli C.. (2018). A fine-scale genetic linkage map reveals genomic regions associated with economic traits in walnut (*Juglans regia*). Plant Breed. 138, 635–646. doi: 10.1111/pbr.12703

[B4] Arus P. Verde I. Sosinski B. Zhebentyayeva T. Abbott A. G. (2012). The peach genome. Tree Genet. &Genomes 8 (3), 531–547. doi: 10.1007/s11295-012-0493-8

[B5] Baro-Montel N. Eduardo I. Usall J. Casals C. Arus P. Teixido N.. (2019). Exploring sources of resistance to brown rot in an interspecific almond x peach population. J. Sci. Food Agric. 99 (8), 4105–4113. doi: 10.1002/jsfa.9640 30784078

[B6] Brizzolara S. Hertog M. Tosetti R. Nicolai B.and Tonutti P. (2018). Metabolic responses to low temperature of three peach fruit cultivars differently sensitive to cold storage. Front. Plant Sci. 9, 706. doi: 10.3389/fpls.2018.00706 29892309 PMC5985494

[B7] Brukental H. Doron-Faigenboim A. Bar-Yaakov I. Harel-Beja R. Attia Z. Azoulay-Shemer T.. (2021). Revealing the genetic components responsible for the unique photosynthetic stem capability of the wild almond *Prunus arabica* (Olivier) meikle. Front. Plant Sci. 12, 779–970. doi: 10.3389/fpls.2021.779970 PMC8657148 34899807

[B8] Calle A. Serradilla M. J. Wunsch A. (2021). QTL mapping of phenolic compounds and fruit colour in sweet cherry using a 6 + 9K SNP array genetic map. Scientia Hortic. 280, 109900. doi: 10.1016/j.scienta.2021.109900

[B9] Cantin C. M. Arus P. Eduardo I. (2018). Identification of a new allele of the dw gene causing brachytic dwarfing in peach. BMC Res. Notes 11, 386. doi: 10.1186/s13104-018-3490-7 29898773 PMC6000960

[B10] Carrasco B. Gonzalez M. Gebauer M. Garcia-Gonza lez R. Maldonado J. Silva H. (2018). Construction of a highly saturated linkage map inJapanese plum (*Prunus salicina* l.) using GBS for SNP marker calling. PloS One 13 (12), 1–14. doi: 10.1371/journal.pone.0208032 PMC6277071 30507961

[B11] Carrier G. Huang Y. F. Le Cunff L. Fournier-Level A. Vialet S. Souquet J. M.. (2013). Selection of candidate genes for grape proanthocyanidin pathway by an integrative approach. Plant Physiol. Biochem. 72, 87–95. doi: 10.1016/j.plaphy.2013.04.014 23684499

[B12] Chagné D. Crowhurst R. N. Pindo M. Thrimawithana A. Deng C. Ireland H.. (2014). The draft genome sequence of European pear (*Pyrus communis* L.‘Bartlett’). PloS One 9 (4), e92644. doi: 10.1371/journal.pone.0092644 24699266 PMC3974708

[B13] Chaney W. Yuanhong H. Rohla C. Monteros M. J. Grauke L. J. (2015). Developing molecular marker resources for pecan. Acta Horticul. 1070, 127–132. doi: 10.17660/ActaHortic.2015.1070.13

[B14] Cockerton H. M. Vickerstaff R. J. Karlstrom A. Wilson F. Sobczyk M. He J. Q.. (2018). Identification of powdery mildew resistance QTL in strawberry (*Fragaria* × *ananassa*). Theor. Appl. Genet. 131, 1995–2007. doi: 10.1007/s00122-018-3128-0 29971472 PMC6096635

[B15] Collard B. C. Y. Jahufer M. Z. Z. Brouwer J. B. Pang E. C. K. (2005). An introduction to markers, quantitative trait loci (QTL) mapping and marker-assisted selection for crop improvement: The basic concepts. Euphytica 142, 169–196. doi: 10.1007/s10681-005-1681-5

[B16] Collard B. C. Y. Mackill D. J. (2008). Marker-assisted selection:an approach for precision plant breeding development twenty-first century. PhilosophicalTransactionsof RoyalSociety Of London 363, 557–572. doi: 10.1098/rstb.2007.2170 PMC2610170 17715053

[B17] Cramer G. R. Ghan R. Schlauch K. A. Tillett R. L. Heymann H. Ferrarini A.. (2014). Transcriptomic analysis of the late stages of grapevine (*Vitis vinifera* cv. Cabernet sauvignon) berry ripening reveals significant induction of ethylene signaling and flavor pathways in the skin. BMC Plant Biol. 14, 370. doi: 10.1186/s12870-014-0370-8 25524329 PMC4312598

[B18] Dhutmal R. R. Mundhe A. G. More A. W. (2018). Molecular marker techniques: A review. Int. J. Curr. Microbiol. Appl. Sci. 6, 816–825.

[B19] El-Sharkawy I. Liang D. Xu K. (2015). Transcriptome analysis of an apple (*Malus* × *domestica*) yellow fruit somatic mutation identifies a gene network module highly associated with anthocyanin and epigenetic regulation. JournalOf Exp. Bot. 66, 7359–7376. doi: 10.1093/jxb/erv433 PMC4765799 26417021

[B20] Fan Z. Tieman D. M. Knapp S. J. Zerbe P. Famula R. Barbey C. R.. (2022). A multiomics frameworkreveals strawberry flavor genes and their regulatory elements. New Phytol. 236 (3), 1089–1107. doi: 10.1111/nph.18416 35916073 PMC9805237

[B21] Frary A. Ozturk S. C. Balik H. I. Balik S. K. Kizilci G. Doganlar S.. (2019). Analysis of European hazelnut (*Corylus avellana*)reveals loci for cultivar improvement and the effects of domestication and selection on nut and kernel traits. Mol. Genet. Genomics 294, 519–527. doi: 10.1007/s00438-018-1527-1 30604072

[B22] Ganie S. A. Wani S. H. Henry R. Hensel G. (2021). Improving rice salt tolerance by precision breeding in a new era. Curr. Opin. Plant Biol. 60, 101996. doi: 10.1016/j.pbi.2020.101996 33444976

[B23] Garcia-Gomez B. E. Salazar J. A. Dondini L. Martinez-Gomez P. Ruiz D. (2019). Identification of QTLs linked to fruit quality traits in apricot (*Prunus armeniaca* l.) and biological validation through gene expression analysis using qPCR. Mol. Breed. 39, 28. doi: 10.1007/s11032-018-0926-7

[B24] Gavicho Uarrota V. Fuentealba C. Hernández I. Defilippi-Bruzzone B. Meneses C. Campos- Vargas R.. (2019). Integration of proteomics and metabolomics data of early and middle season hass avocados under heat treatment. Food Chem. 289, 512–521. doi: 10.1016/j.foodchem.2019.03.090 30955643

[B25] Guo J. Cao K. Deng C. Li Y. Zhu G. Fang W.. (2020). An integrated peach genome structural variation map uncovers genes associated with fruit traits. Genome Biol. 21, 258. doi: 10.1186/s13059-020-02169-y 33023652 PMC7539501

[B26] Halasz J. Kodad O. Galiba G. M. Skola I. Ercisli S. Ledbetter C. A.. (2019). Genetic diversity is preserved among strongly differentiated and geographically diverse almond germplasm:an assessment by simple sequence repeat markers. Tree Genet. &Genomes 15, 12. doi: 10.1007/s11295-019-1319-8

[B27] Hale I. Melo A. T. O. Gustafson H. (2018). Sex-linked molecular markers for two cold-hardy kiwifruit species, *Actinidia arguta* and a.kolomikta. Eur. J. Hortic. Sci. 83 (4), 236–246. doi: 10.17660/eJHS.2018/83.4.4

[B28] Han H. Oh Y. Kim K. Oh S. Cho S. Kim Y.. (2019). Integrated genetic linkage maps for Korean pears (*Pyrus* hybrid) using GBS-based SNPs and SSRs. Horticul. Environment Biotechnol. 60, 779–786. doi: 10.1007/s13580-019-00171-3

[B29] Hayward A. C. Tollenaere R. Morgan J. D. Batley J. (2015). Molecular marker applications in plants. Methods Mol. Biol. 1245, 13–27. doi: 10.1007/978-1-4939-1966-6_2 25373746

[B30] Hirakawa H. Shirasawa K. Kosugi S. Tashiro K. Nakayama S. Yamada M.. (2014). Dissection of the octoploid strawberry genome by deep sequencing of the genomes of fragaria species. DNA Res. 21, 169–181. doi: 10.1093/dnares/dst049 24282021 PMC3989489

[B31] Hossain M. R. Natarajan S. Kim H. T. Jesse D. M. I. Lee C. G. Park J. I.. (2019). High density linkage map construction and QTL mapping for runner production in allo-octoploid strawberry fragaria x *ananassa* based on dd RAD-seq derived SNPs. Sci. Rep. 9, 3275. doi: 10.1038/s41598-019-39808-9 30824841 PMC6397268

[B32] Houmanat K. Abdellah K. Hssaini L. Razouk R. Hanine H. Jaafary S.. (2020). Molecular diversity in walnut(*Juglans regia* l.)among two major areas in Morocco in contrast with foreign varieties. Int. J. Fruit Sci. 21 (1), 180–192. doi: 10.1080/15538362.2020.1862734

[B33] Huang D. Zhao Y. Cao M. Qiao L. Zheng Z. (2016). Integrated systems biology analysis of transcriptomes reveals candidate genes for acidity control in developing fruits of sweet orange (*Citrus sinensis* l. osbeck). Front. Plant Sci. 7, 486. doi: 10.3389/fpls.2016.00486 27092171 PMC4824782

[B34] Igarashi M. Hatsuyama Y. Harada T. Fukasawa-Akada T. (2015). Biotechnology and apple breeding in Japan. Breed. Sci. 66, 18–33. doi: 10.1270/jsbbs.66.18 PMC4780799 27069388

[B35] Jaillon O. Aury J. M. Noel B. Policriti A. Clepet C. Casagrande A.. (2007). The grapevine genome sequence suggests ancestral hexaploidization in major angiosperm phyla. Nature 449 (7161), 463–467. doi: 10.1038/nature06148 17721507

[B36] Jain H. K. Kharkwal M. C. (Eds.) (2004). Plant breeding mendelian to molecular approaches (New Delhi, India: Narosa publishing House), 810.

[B37] Jan A. (2016). Morpho-genetic diversity studies in sandy pear (Pyrus pyrifolia.Nakai) (Shalimar Campus Srinagar: Faculty of Horticulture SKUAST-Kashmir), 98.

[B38] Jiang J. Fan X. Zhang Y. Tang X. Li X. Liu C.. (2020). Construction of a high-density genetic map and mapping of firmness in grapes (*Vitis vinifera* l.) based on whole genome resequencing. Int. J. Mol. Sci. 21 (3), 797. doi: 10.1038/s41438-019-0215-6 31991832 PMC7037167

[B39] Jiang F. Zhang J. Wang S. Yang L. Luo Y. Gao S.. (2019). The apricot(*Prunus armeniaca* l.) genome elucidates rosaceae evolution and beta-carotenoid synthesis. Horticul. Res. 6, 128. doi: 10.3390/ijms21030797 PMC6861294 31754435

[B40] Lenz R. R. Dai W. (2017). Mapping X-disease phytoplasma resistance in *Prunus virginiana* . Front. Plant Sci. 8, 2057. doi: 10.3389/fpls.2017.02057 29238359 PMC5712551

[B41] Le Roux P. M. F. Christen D. Duffy B. Tartarini S. Dondini L. Yamamoto T.. (2012). Redefinition of the map position and validation of a major quantitative trait locus for fire blight resistance of the pear cultivar “ harrow sweet” (*Pyrus communis* l.). Plant Breed. 131, 656–664. doi: 10.1111/j.1439-0523.2012.02000.x

[B42] Lewers K. S. Castro P. Hancock J. F. Weebadde C. K. Die J. V. Rowland L. J. (2019). Evidence of epistatic suppression of repeat fruiting in cultivated strawberry. BMC Plant Biol. 19, 386. doi: 10.1186/s12870-019-1984-7 31488054 PMC6729047

[B43] Li W. Liu L. Wang Y. Zhang Q. Fan G. Zhang S.. (2020). Genetic diversity, population structure, and relationships of apricot (Prunus) based on restriction site-associated DNA sequencing. Horticult. Res. 7. doi: 10.1038/s41438-020-0284-6 PMC7192913 32377359

[B44] Lian X. Zhao X. Zhao Q. Wang G. Li Y. Hao Y. (2021). MdDREB2A in apple is involved in the regulation of multiple abiotic stress responses. Hortic. Plant J. 7 (3), 197–208. doi: 10.1016/j.hpj.2021.03.006

[B45] Lim S. Lee J. Lee H. J. Park K. H. Kim D. S. Min S. R.. (2016). The genetic diversity among strawberry breeding resources based on SSRs. Scientia Agricola 74 (3), 226–234. doi: 10.1590/1678-992X-2016-0046

[B46] Liu W. Shahid M. Q. Bai L. Lu Z. Chen Y. Jiang L.. (2015). Evaluation of genetic diversity and development of a core collection of wild rice(*Oryza rufipogon* griff.)populations in China. PloS One 10 (12), e0145990. doi: 10.1371/journal.pone.0145990 26720755 PMC4703137

[B47] Liu J. Sui Y. Chen H. Liu Y. Liu Y. (2018). Proteomic analysis of kiwifruit in response to the postharvest pathogen, *Botrytis cinerea* . Front. Plant Sci. 9, 158. doi: 10.3389/fpls.2018.00158 29497428 PMC5818428

[B48] Lozano L. Iglesias I. Micheletti D. Troggio M. Kumar S. Volz R. K.. (2014). Feasibility of genome-wide association analysis using a small single nucleotide polymorphism panel in an apple breeding population segregating for fruit skin color. J. Am. Soc. Hortic. Sci. 139 (6), 619–526. doi: 10.21273/JASHS.139.6.619

[B49] Marrano A. Britton M. Zaini P. A. Zimin A. V. Workman R. E. Puiu D.. (2020). High-quality chromosome-scale assembly of the walnut (Juglans regia l.) reference genome. Gigascience 9 (5), giaa050. doi: 10.1093/gigascience/giaa050 32432329 PMC7238675

[B50] Marimon N. Luque J. Arus P. Eduardo I. (2020). Fine mapping and identification of candidate genes for the peach powdery mildew resistance gene *Vr3* . Horticul. Res. 7, 175. doi: 10.1038/s41438-020-00396-9 PMC7603514 33328431

[B51] Marrano A. Sideli G. M. Leslie C. A. Cheng H. Neale D. B. (2019). Deciphering of the genetic control of phenology, yield, and pellicle color in Persian walnut (*Juglans regia* l.). Front. Plant Sci. 10, 1140. doi: 10.3389/fpls.2019.01140 31616449 PMC6764078

[B52] Martinez-Garcia P. J. Crepeau M. W. Puiu D. Gonzalez-Ibeas D. Whalen J. Stevens K. A.. (2016). The walnut(*Juglans regia*) genome sequence reveals diversity in genes coding for the biosynthesis of non-structural polyphenols. Plant J. 87 (5), 507–532. doi: 10.1111/tpj.13207 27145194

[B53] Martinez-Zapater J. M. Carmona M. J. DiazRiquelme J. Fernandez L. Lijavetzky D. (2010). Grapevinegenetics after the genome sequence: Challenges and limitations. Aust. J. Grape Wine Res. 16, 33–46. doi: 10.1111/j.1755-0238.2009.00073.x

[B54] Mas-Gomez J. Cantin C. M. Moreno M. A. Prudencio A. S. GomezAbajo M. Bianco L.. (2021). Exploring genome-wide diversity in the national Peach(*Prunus persica*) germplasm collection at CITA (Zaragoza, Spain). Agronomy 11, 481. doi: 10.3390/agronomy11030481

[B55] Mathiazhagan M. Chidambara B. Hunashikatti L. R. Ravishankar K. V. (2021). Genomic approaches for improvement of tropical fruits: fruit quality, shelf life and nutrient content. Genes 12 (12), 1881. doi: 10.3390/genes12121881 34946829 PMC8701245

[B56] Mehlenbacher S. A. (1995). Classical and molecular approaches to breeding fruit and nutcrops for diseases resistance. Hortic. Sci. 30, 466–477.

[B57] Montanari S. Perchepied L. Renault D. Frijters L. Velasco R. Horner M.. (2016). A QTL detected in an interspecific pear population confers stable fire blight resistance across different environments and genetic backgrounds. Mol. Breed. 36, 47. doi: 10.1007/s11032-016-0473-z

[B58] Mori G. D. Falchi R. Testolin R. Bassi D. Savazzini F. Dondini L.. (2019). Resistance to sharka in apricot:Comparison of phase-reconstructed resistant and susceptible haplotypes of ‘Lito’chromosome 1 and analysis of candidate genes. Front. Plant Sci. 10, 1576. doi: 10.3389/fpls.2019.01576 31867032 PMC6905379

[B59] Muthamilarasan M. Singh N. K. Prasad M. (2019). Multi-omics approaches for strategic improvement of stress tolerance in underutilized crop species: a climate change perspective. Advanced Genet. 103, 1–38. doi: 10.1016/bs.adgen.2019.01.001 30904092

[B60] Nadeem M. A. Nawaz M. A. Shahid M. Q. Dogan Y. Comertpay G. Yildiz M.. (2018). DNA Molecular markers in plant breeding:current status and recent advancements in genomic selection and genome editing. Biotechnol. Biotechnol. Equip. 32 (2), 261–285. doi: 10.1080/13102818.2017.1400401

[B61] Naeem M. Ghouri F. Shahid M. Q. Iqbal M. Baloch F. S. Chen L.. (2015). Genetic diversity in mutated and non-mutated rice varieties. Genet. Mol. Res. 14 (4), 17109–17123. doi: 10.4238/2015.December.16.11 26681058

[B62] Nawaz M. A. Baloch F. S. Rehman H. M. Le B. Akther F. Yang S. H.. (2016). Development of a competent and trouble free DNA protocol for downstream genetic analysis in glycine species. Turkish J. Agriculture-Food Sci. Technol. 4 (8), 700–705. doi: 10.24925/turjaf.v4i8.700-705.788

[B63] Nawaz M. A. Rehman H. M. Baloch F. S. Ijaz B. Ali M. A. Khan I. A.. (2017). Genome and transcriptome-wide analysis of cellulose synthase gene superfamily in soyabean. J. Plant Physiol. 215, 163–175. doi: 10.1016/j.jplph.2017.04.009 28704793

[B64] Nawaz M. A. Sadia B. Awan F. S. Zia M. A. Khan I. A. (2013). Genetic diversity in hyper glucose oxidase producing *Aspergillus niger* UAF mutants by using molecular markers. Int. J. Agric. Biotechnol. 15 (2), 362–366.

[B65] Nawaz M. A. Yang S. H. Rehman H. M. Baloch F. S. Lee J. D. Park J. H.. (2017). Genetic diversity and population structure of Korean wild soyabean(*Glycine soja* sieb. and zucc.) inferred from microsatellite markers. Biochem. Systematics Ecol. 71, 87–96. doi: 10.1016/j.bse.2017.02.002

[B66] Nishio S. Terakami S. Matsumoto T. Yamamoto T. Takada N. Kato H.. (2018). Identification of QTLs for agronomic traits in Japanese chestnut (*Castanea crenata* sieb.et zucc.) breeding. Horticul. J. 87 (1), 43–54. doi: 10.2503/hortj.OKD-093

[B67] Nunziata A. Ruggieri V. Petriccione M. Masi L. D. (2020). Single nucleotide polymorphisms as practical molecular tools to support European chestnut agrobiodiversity management. Int. J. Mol. Sci. 21, 4805. doi: 10.3390/ijms21134805 32646057 PMC7370276

[B68] Oh S. Lee M. Kim K. Han H. Won K. Kwack Y.. (2019). Genetic diversity of kiwifruit(*Actinidia* spp.), including Korean native *A.arguta*, using single nucleotide polymorphisms derived from genotyping-by-sequencing. Horticul. Biotechnol. Biotechnol. 60 (1), 105–114. doi: 10.1007/s13580-018-0106-z

[B69] Oliveira L. S. S. Pimenta L. V. A. Guimaraes L. M. S. deSouza P. V. D. Bhering L. L. Alfenas A. C. (2020). Resistance of kiwifruit cultivars to *Ceratocystis* wilt:an approach considering the genetic diversity and variation in aggressiveness of the pathogen. Plant Pathol. 70 (2), 349–357. doi: 10.1111/ppa.13305

[B70] Öztürk S. C. Balık H. İ. Balık S. K. Kızılcı G. Duyar Ö. Doğanlar S.. (2017). Molecular genetic diversity and association mapping of nut and kernel traits in Slovenian hazelnut (*Corylus avellana*) germplasm. Tree Genet. Genomics 13 (5), 1–10.

[B71] Paizila A. Kafkas S. Ziya-Motalebipour E. Acar I. Turemis N. (2019). Construction of an almond genetic linkage map using F1 population ‘Gulcan -2’x ‘Lauranne’ by SSR markers. Acta Horticul. 1242, 543–548. doi: 10.17660/ActaHortic.2019.1242.79

[B72] Patzak J. Henychová A. Paprštein F. Sedlák J. (2019). Evaluation of genetic variability within sweet cherry (Prunus avium L.) genetic resources by molecular SSR markers. Acta Sci. Pol. Hortorum Cultus 18, 157–165. doi: 10.24326/asphc.2019.3.15

[B73] Patzak J. Henychova A. Paprstein F. Sedlak J. (2020). Evaluation of s-incompatibility locus, genetic diversity and structure of sweet cherry (*Prunus avium* l.) genetic resources by molecular methods and phenotypic characteristics. J. Hortic. Sci. Biotechnol. 95 (1), 84–92. doi: 10.1080/14620316.2019.1647798

[B74] Peace C. P. Bianco L. Troggio M. vandeWeg E. Howard N. P. Cornille A.. (2019). Apple whole genome sequences: recent advances and new prospects. Horticul. Res. 6, 59. doi: 10.1038/s41438-019-0141-7 PMC6450873 30962944

[B75] Rasool A. (2018). Morpho-molecular characterization of apple (Malusxdomestica.Borkh) germplasm (Shalimar Campus Srinagar: Faculty of Horticulture SKUAST-Kashmir), 121.

[B76] Rasouli M. Fatahi R. Zamani Z. Imani A. Martinez-Gomez P. (2018). Identification of DNA markers linked to blooming time in almond. J. Nuts 92 (2), 105–122. doi: 10.22034/JON.2018.543679

[B77] Raza S. Farooqi S. Mubeen H. (2015). Role of molecular markers and their significance. Am. J. Pharm. Health Res. 3 (12), 23–30.

[B78] Riaz S. Lorenzis G. D. Velasco D. Koehmstedt A. Maghradze D. Bobokashvili Z.. (2018). Genetic diversity analysis of cultivated and wild grapevine (*Vitis vinifera* l.) accessions around the Mediterranean basin and central Asia. BMC Plant Biol. 18, 137. 29945553 10.1186/s12870-018-1351-0 PMC6020434

[B79] Salazar J. A. Pacheco I. Zapata P. Shinya P. Ruiz D. Martinez-Gomez P.. (2020). Identification of loci controlling phenology, fruit quality and post-harvest quantitative parameters in Japanese plum(*Prunus saliciana* lindl.). Postharvest Biol. Technol. 169, 111292. doi: 10.1016/j.postharvbio.2020.111292

[B80] Samantara K. Reyes V. P. Agrawal N. Mohapatra S. R. Jena K. K. (2021). Advances and trends on the utilization of multi-parent advanced generation intercross (MAGIC) for crop improvement. Euphytica 217 (10), 1–22.

[B81] Sanchez-Sevilla J. F. Horvath A. Botella M. A. Gaston A. Folta K. Kilian A.. (2015). Diversity arrays technology (DarT) marker platforms for diversity analysis and linkage mapping in a complex crop, the octoploid strawberry (*Fragaria* x *ananassa*). PloS One 10 (12), e0144960. doi: 10.1371/journal.pone.0144960 26675207 PMC4682937

[B82] Santos C. Nelson C. D. Zhebentyayeva T. Machado H. Gomes-Laranjo J. Costa R. L. (2017). First interspecific genetic linkage map for *Castanea sativa* x *Castanea crenata* revealed QTLs for resistance to *Phytophthora cinnamomi* . PloS One 12 (9), e0184381. doi: 10.1371/journal.pone.0184381 28880954 PMC5589223

[B83] Santos C. Zhebentyayeva T. Serrazina S. Nelson C. D. Costa R. (2015). Development and characterization of EST-SSR markers for mapping reaction to phytophthora cinnamomi in castanea spp. Scientia Hortic. 194, 181–187. doi: 10.1016/j.scienta.2015.07.043

[B84] Shulaev V. Sargent D. J. Crowhurst R. N. Mockler T. C. Folkerts O. Delcher A. L.. (2011). The genome of woodland strawberry (*Fragaria vesca*). Nat. Genet. 43, 109–116. doi: 10.1038/ng.740 21186353 PMC3326587

[B85] Syed S. (2019). Identification of selected apple clonal rootstocks through molecular and morphological approaches (Shalimar Campus Srinagar: Faculty of Horticulture SKUAST-Kashmir).

[B86] Terakami S. Adachi Y. Takeuchi Y. Takada N. Nishio S. Saito T.. (2021). Development of an SSR marker set for efficient selection for resistance to black spot disease in pear breeding. Breed. Sci. 71 (2), 240–252. doi: 10.1270/jsbbs.20136 34377072 PMC8329887

[B87] Thottappilly G. Magonouna H. D. Omitogun O. G. (2000). The use of DNA markers for rapid improvement of crops in Africa. Afr. Crop Sci. J. 8, 99–108. doi: 10.4314/acsj.v8i1.27720

[B88] Thurow L. B. Raseira M. D. C. B. Bonow S. Arge L. W. P. Castro C. M. (2016). Population genetic analysis of Brazilian peach breeding germplasm. Rev. Bras. Fruiticul. 39 (5), 166. doi: 10.1590/0100-29452017166

[B89] Tiwari J. K. Singh B. P. Gopal J. Poonam P. Patil V. U. (2013). Molecular characterization of the Indian andigena potato core collection using microsatellite markers. Afr. J. Biotechnol. 12 (10), 1025–1033.

[B90] Velasco R. Zharkikh A. Affourtit J. Gardiner S. E. Skolnick M. Egholm M.. (2010). The genome of the domesticated apple (*Malus*x*domestica* borkh.). Nat. Genet. 42 (10), 833–839. doi: 10.1038/ng.654 20802477

[B91] Wang Y. Ghouri F. Shahid M. Q. Naeem M. Baloch F. S. (2017). The genetic diversity and population structure of wild soyabean evaluated by chloroplast and nuclear gene sequences. Biochem. Systematics Ecol. 71, 170–178. doi: 10.1016/j.bse.2017.02.008

[B92] Wang Y. Shahid M. Q. Ghouri F. Wang Y. Huang H. (2015). Evaluation of the geographical pattern of genetic diversity of glycine soja and *Glycine max* based on four single copy nuclear gene loci:for conservation of soyabean germplasm. Biochem. Systematics Ecol. 62, 229–235. doi: 10.1016/j.bse.2015.09.006

[B93] Wang J. Zhang K. Zhang X. Yan G. Zhou Y. Feng L.. (2015). Construction of commercial sweet cherry linkage maps and QTL analysis for trunk diameter. PloS One 10 (10), e0141261. doi: 10.1371/journal.pone.0141261 26516760 PMC4627659

[B94] Wani S. H. Choudhary M. Barmukh R. Bagaria P. K. Samantara K. Razzaq A.. (2022). Molecular mechanisms, genetic mapping, and genome editing for insect pest resistance in field crops. Theor. Appl. Genet., 1–21. doi: 10.1007/s00122-022-04060-9 PMC9729161 35267056

[B95] Wani S. H. Samantara K. Razzaq A. Kakani G. Kumar P. (2022). Back to the wild: mining maize (*Zea mays* l.) disease resistance using advanced breeding tools. Mol. Biol. Rep. 22, 1–17. 10.1007/s11033-021-06815-x 35064401

[B96] Wohner T. W. Emeriewen O. F. Wittenberg A. H. J. Schneiders H. Vrijenhoek I. Halasz J.. (2021). The draft chromosome-level genome assembly of tetraploid ground cherry (*Prunus fruticosa* pall.) from long reads. Genomics 113, 4173–4183. doi: 10.1016/j.ygeno.2021.11.002 34774678

[B97] Wu B. H. Cao Y. G. Guan L. Xin H. P. Li J. H. Li S. H. (2014). Genome-wide transcriptional profiles of the berry skin of two red grape cultivars (*Vitis vinifera*) in whichanthocyanin synthesis is sunlight-dependent or -independent. PloS One 9, e105959. doi: 10.1371/journal.pone.0105959 25158067 PMC4144973

[B98] Wu W. Chen F. Yeh K. Chen J. (2018). ISSR analysis of genetic diversity and structure of plum varieties cultivated in southern china. Biology 8, 2. 30577627 10.3390/biology8010002 PMC6466324

[B99] Wu H. Ma T. Kang M. Ai F. Zhang J. Dong G.. (2019). A high –quality *Actinidia chinensis* (kiwifruit) genome. Horticul. Res. 6, 117. doi: 10.1038/s41438-019-0202-y PMC6804796 31645971

[B100] Xiao Y. Wang C. Tian Y. Yang S. Shen J. Liu Q.. (2019). Candidates responsible for dwarf pear phenotype as revealed by comparative transcriptome analysis. Mol. Breed. 39, 1. doi: 10.1007/s11032-018-0907-x

[B101] Xie M. Huang Y. Zhang Y. Wang X. Yang H. Yu O.. (2013). Transcriptome profiling of fruit development and maturation in Chinese white pear (*Pyrus bretschneideri* rehd). BMC Genomics 14, 823. doi: 10.1186/1471-2164-14-823 24267665 PMC4046828

[B102] Yang C. Sha G. Wei T. Ma B. Li C. Li P.. (2020). Linkage map and QTL mapping of red flesh locus in apple using a R1R1 x R6R6 population. Hortic. Plant J. 7 (5), 393–400. doi: 10.1016/j.hpj.2020.12.008

[B103] Yokomi R. Delgado J. K. Unruh T. R. Barcenas N. M. Garczynski S. F. Walse S.. (2021). Molecular advances in larval fruit moth identification to facilitate fruit export from Western united states under systems approaches. Ann. Entomol. Soc. America 115 (1), 105–112. doi: 10.1093/aesa/saab040

[B104] Zaini P. A. Feinberg N. G. Grilo F. S. Saxe H. J. Salemi M. R. Phinney B. S.. (2020). Comparative proteomic analysis of walnut (*Juglans regia* l.) pellicle tissues reveals the regulation of nut quality attributes. Life 10 (12), 314. doi: 10.1139/cjps-2018-0177 33261033 PMC7760677

[B105] Zhang J. Sun H. Yang L. Jiang F. Zhang M. Wang Y. (2019). Construction of a high-density linkage map and QTL analysis for pistil abortion in apricot (*Prunus armeniaca* l.). Can. J. Plant Sci. 99, 599–610. doi: 10.1139/cjps-2018-0177

[B106] Zhang C.-x. Tian Y. Cong Ph (2015). Proteome analysis of pathogenresponsive proteins from apple leaves induced by the alternaria blotch *Alternaria alternata* . PloS One 10, e0122233. 26086845 10.1371/journal.pone.0122233 PMC4472855

[B107] Zhang C. Yao X. Ren H. Chang J. Wu J. Shao W.. (2020). Characterization and development of genomic SSRs in Pecan (*Carya illinoinensis*). Forests 11, 61. doi: 10.3390/f11010061

[B108] Zhao H. Ju Y. Jiang J. Min Z. Fang Y. Liu C. (2019). Downy mildew resistance identification and SSR molecular marker screening of different grape germplasm resources. Scientia Hortic. 252, 212–221. doi: 10.1016/j.scienta.2019.03.025

[B109] Zhen Q. Fang T. Peng Q. Liao L. Zhao L. Owiti A.. (2018). Developing gene-tagged molecular markers for evaluation of genetic association of apple SWEET genes with fruit sugar accumulation. Horticul. Res. 5, 14. doi: 10.1038/s41438-018-0024-3 PMC5859117 29581882

[B110] Zheng S. Hao Y. Fan S. Cai J. Chen W. Li X.. (2021). Metabolomic and transcriptomic profiling provide novel insights into fruit ripening and ripening disorder caused by 1-MCP treatments in papaya. Int. JournalOf Mol. Sci. 22, 916. doi: 10.3390/ijms22020916 PMC7831311 33477620

[B111] Zhu Y. Yin Y. Yang K. Li J. Sang Y. Huang L.. (2015). Construction of high-density genetic map using specific length amplified fragment markers and identification of a quantitative trait locus for resistance in walnut (*Juglans regia* l.). BMC Genomics 16 (1), 1–13. doi: 10.1186/s12864-015-1822-8 26283231 PMC4539690

